# Adverse Drug Reactions After Administration of Emodepside/Praziquantel (Profender®) in an MDR1-Mutant Australian Shepherd Dog: Case Report

**DOI:** 10.3389/fvets.2019.00296

**Published:** 2019-09-06

**Authors:** Daniela Gaens, Carola Leithäuser, Melanie Hamann, Joachim Geyer

**Affiliations:** ^1^Faculty of Veterinary Medicine, Institute of Pharmacology and Toxicology, Justus Liebig University Giessen, Giessen, Germany; ^2^Animal Clinic Norderstedt, Evidensia Tierärztliche Klinik Für Kleintiere, Norderstedt, Germany

**Keywords:** Profender, MDR1 (ABCB1) gene, emodepside, MDR1 mutation, dog, adverse drug reaction, drug intolerance, neurological toxicity

## Abstract

A 3-year-old male Australian Shepherd was presented with signs of neurological toxicity following the administration of Profender® at the recommended dosage. Unfortunately, the owner had received the product from a veterinarian without any further instructions on fasting as recommended by the manufacturer, so the dog was fed prior to Profender® administration. Neurological toxicity included generalized tremor, agitation and panting, and required hospitalization of the dog. All neurological signs resolved after symptomatic treatment within 24 h and the dog was discharged without the need for further medication. MDR1 genotyping revealed a homozygous mutation of the MDR1 gene, which is normally important to prevent brain penetration of emodepside by an efflux-based transport mechanism at the blood brain barrier. This case indicates that Profender® can lead to serious, but transient neurological toxicity in dogs with homozygous MDR1 mutation even at therapeutic dosage, in particular when fasting recommendations are disregarded. Therefore, the case report highlights both the importance of MDR1 genotyping in predisposed dog breeds as well as strict compliance with fasting recommendations around the time of Profender® administration.

## Introduction

The cyclic octadepsipeptide emodepside (EMO), a metabolite of the fungus *Mycellia sterilia*, is used clinically to treat different kinds of gastrointestinal nematodes in cats and dogs (1–3). In dogs, it is administered orally as a modified-release tablet (Profender®) in combination with praziquantel (PZQ) at a minimal therapeutic dosage of 1 mg/kg EMO and 5 mg/kg PZQ (4). Dogs should be fasted prior to drug application, since serum concentrations of EMO increase when dogs are fed at the time of treatment (5).

In parasitic nematodes, EMO interferes with latrophilin receptors (6, 7) and SLO-1 calcium-activated potassium channels (8), leading to paralysis of the pharynx and body-wall muscles, and finally resulting in inhibition of locomotion, feeding, and egg-laying (7, 9, 10). In contrast, EMO has a low acute toxicity in target animals (5, 11) and normally does not enter the brain of vertebrate species due to drug efflux at the blood-brain barrier via the multidrug resistance (MDR1, syn. ABCB1) efflux transporter (12). However, dogs with a homozygous mutation of the MDR1 gene [referred to as nt230(del4) MDR1 mutation (13)] are presumed to show increased drug brain penetration and often suffer from neurological toxicity after application of Profender®, in particular when they are not strictly fasted prior to drug application (14). Dogs predisposed for the MDR1 mutation mostly come from the Collie, Australian Shepherd, Shetland Sheepdog, Longhaired Whippet, and White Shepherd breeds (15).

The current case report describes an MDR1 mutant Australian Shepherd with serious adverse reactions shortly after the administration of Profender®.

## Case presentation

A 3-year-old male Australian Shepherd (weight: 17 kg) was presented at the Veterinary Clinic Norderstedt with generalized tremor, agitation, and panting, which occurred after oral treatment with two Profender® tablets for medium dogs (10 mg EMO plus 50 mg PZQ). The owner had received the product from a veterinarian without any further instructions on fasting, so the dog received a handful (30 g) of dry dog food (Bosch Active) about half an hour before the Profender® administration. Two and a half hours after drug application, the dog showed progressive tremor and severe panting. Furthermore, he developed excessive drooling and salivation and became ataxic.

Therefore, the dog was presented at a veterinary clinic immediately. Initial physical examination revealed a rectal temperature of 40.2°C (104.4°F), heart rate of 136/min, red mucous membranes and a normal capillary refill time of 1 s. The dog showed agitated general condition, panting, and generalized tremor, but was able to stand and walk on his own. Results of further neurological examination were unremarkable. As the patient's condition was stable, the treating veterinarian decided to perform no further clinical examinations (e.g., blood or urine analysis) and symptomatic treatment was initiated.

The dog was admitted to the intensive care unit and possible seizure activity, body temperature, and intravenous fluid therapy were monitored closely. An IV catheter (Vasofix® Braunüle®, Braun Melsungen) was placed in the right cephalic vein. The patient obtained an intravenous lipid emulsion (Lipofundin® MCT/LCT 20%, Braun Melsungen) as an IV bolus of 2.0 mL/kg followed by an infusion of 210 mL for the next hour. IV fluid therapy (Sterofundin® Iso, Braun Melsungen) was also administered at a maintenance rate of 10.0 mL/kg for 3 h, followed by 2.0 mL/kg/h until the next day. Additionally, 1.0 mg/kg diazepam (Ziapam®, 5 mg/ml, Ecuphar) was administered IV to treat the generalized tremor. Since no clinical improvements were noted, 0.2 mL acepromazine (Vetranquil® 1%, Ceva) were administered IM. Subsequently, the patient's condition improved and the generalized tremor changed to a slight trembling within an hour. Body temperature normalized after 4 h to 38.1°C (100.6°F).

One day following admission, no abnormalities were detected at the physical examination. The dog was alert, mucous membranes were pink and moist, heart rate was 84/min, the respiratory rate was 24/min, the rectal temperature was 38.6°C (101.5°F), the abdomen was slightly tensed and rectal examination was unremarkable. No defecation was observed during the hospitalization, but appetite and urination were normal. Neurological examination was also unremarkable and the dog was discharged to his owner without further medication. The owner reported that the dog vomited multiple times the week after discharge. Apart from that, the dog's behavior had recovered completely.

Since Australian Shepherds are predisposed to carry the nt230(del4) MDR1 mutation, which is associated with increased susceptibility against several MDR1 reactive drugs, including EMO, MDR1-genotyping was performed (TransMIT GmbH, Giessen, Germany). The dog showed the homozygous mutant MDR1^−/−^ genotype, resulting in a complete loss of MDR1-mediated drug efflux at the blood-brain barrier.

## Discussion

Profender® tablets, containing EMO and PZQ, are used for the treatment of nematode and cestode infestations and infections in dogs and are generally well-tolerated. EMO is a broad-spectrum anti-parasitic drug and is effective even against multiresistant parasite strains, which suggests a novel mode of action (2, 3, 16–19). Potential targets for EMO in invertebrates are SLO-1 calcium-activated potassium channels (8, 20, 21), G-protein-coupled latrophilin-like (LAT-1) receptors (6, 7), and gamma-aminobutyric acid (GABA) receptors (1) while the latter seem to be of minor importance (18, 22). In contrast, pharmacodynamic interactions of EMO with vertebrate central nervous system (CNS) receptors are scarcely understood. Nevertheless, it is well-established that EMO has a low toxicity in mammals (11, 23–27). In the present case, however, there is a suspected adverse reaction related to the administration of Profender®. As PZQ has a wide margin of safety and low acute toxicity (28, 29), it is likely that neurological toxicity is provoked by EMO and not by PZQ in this case.

There are two factors known that limit the therapeutic safety of Profender® in dogs: (I) feeding around the time of drug administration and (II) mutation of the MDR1 drug efflux carrier at the blood-brain barrier. Both are related to the pharmacokinetics and brain penetration of EMO. Profender® modified-release tablets have an intended prolonged release period of the active substance in fasted animals. However, feeding around the time of drug administration accelerates the release of the active substance and thus leads to increased plasma drug levels. Studies analyzing the pharmacokinetics of EMO and PZQ after Profender® administration revealed significantly higher C_max_ values in dogs fed around the time of treatment compared to fasted dogs (5). Therefore, overnight fasting of a dog is recommended when Profender® treatment is intended for the next morning and food should not be made available until 4 h after treatment (4). As in the current case, in practice this recommendation is often disregarded, either due to ignorance or because drug application together with food is better accepted by dogs. However, whereas the increase in plasma concentration at a non-fasted condition seems to be covered by the therapeutic index in MDR1 intact dogs, this does not apply for MDR1 mutant dogs (14). In these dogs, drug efflux by the MDR1 efflux transporter is abolished due to a 4-bp deletion in exon 4 of the MDR1 gene, commonly referred to as nt230(del4) MDR1 mutation (13). Consequently, dogs homozygous for this mutation (MDR1^−/−^ dogs) are completely deficient for MDR1-mediated drug efflux. Dogs from several breeds are predisposed for this mutation including Collie, Australian Shepherd, Shetland Sheepdog, Longhaired Whippet, White Shepherd, and some others (15). In an *mdr1*-mutant mouse model, we recently showed significantly increased brain penetration of EMO in the absence of MDR1 in the blood-brain barrier. The brain concentrations of EMO in the *mdr1*-deficient mice were almost twice as high as the corresponding plasma concentration, whereas in *mdr1*-intact animals, no relevant EMO concentrations were detectable in the brain (12). In the *mdr1*-mutant mice, neurological toxicity indicated by ataxia, hyperkinesia, and behavioral changes was detected already at 1 mg/kg oral dosage, whereas *mdr1*-intact mice did not show any adverse drug reactions. This indicates that the MDR1 drug efflux at the blood-brain barrier normally prevents neurological toxicity after EMO application. A retrospective study on 55 dogs with adverse drug reactions after Profender® treatment revealed the MDR1^−/−^ mutant genotype in most of the cases. Ataxia, salivation, tremor, panting, and agitation were the most prominent symptoms in these dogs (14). The Australian Shepherd dog in the current case had the MDR1^−/−^ deficient genotype as well. Therefore, it is very likely that neurological toxicity, including tremor, panting, salivation, ataxia, hyperthermia, and agitation, were provoked by increased drug penetration into the brain due to MDR1 deficiency.

Altogether, there is increasing evidence that dogs with homozygous MDR1 mutation and non-fasted administration of Profender® tablets are particularly prone to adverse drug reactions involving the nervous system. In this case, two factors are combined that have been shown to limit the therapeutic safety of Profender®. Unfortunately, this scenario was not evaluated during drug approval. The only pre-clinical studies that have been performed showed either accelerated drug release and absorption in non-fasted dogs, or lower margin of safety in MDR1 mutant Collie dogs that were fasted as recommended (4, 5). Therefore, occurrence of neurological toxicity in the current case explicitly does not conflict with these pre-clinical target animal studies. According to the Guidelines on Pharmacovigilance for Medical Products for Veterinary Use [volume 9B of The Rules Governing Medicinal Products in the European Union (30)], a probable causal relationship exists between the administration of Profender® and the adverse reactions in the case presented here. The description of the clinical phenomena in this case is consistent with the known pharmacology and toxicology of Profender®. Additionally, there is a reasonable association in time and there are almost no other equally plausible explanations. Furthermore, previous knowledge of similar reports exist in literature (14) and the adverse events are described in the Summary of Product Characteristics (SPC) (4) as well. Additionally, in accordance with the Directive 2001/82/EC (31), adverse reactions are serious in this case. Both mentioned factors known to limit the therapeutic safety of Profender® were disregarded in this case, despite strict recommendation and information in the SPC. Therefore, this case report indicates that in practice, the use of Profender® still leads to adverse drug reactions in MDR1 mutant dogs, mainly due to ignorance or poor information of dog owners about the strict fasting regime required around the time of drug administration.

In general, MDR1-genoypting in dogs from predisposed breeds is recommended prior to administration of MDR1 reactive drugs, including EMO. Additionally, fasting recommendations of Profender® should be strictly followed and dog owners should always be informed, in particular in dogs with homozygous MDR1-mutation.

As specified in the SPC, signs of neurological disorders may be more severe in MDR1 mutant dogs, but symptoms are transient and self-resolving without any treatment (4). However, adverse reactions of the Australian Shepherd in the current case were serious and very distressing to both animal and owner, in a way that was not expected from the SPC statement. This is particularly significant, since there is no antidote or treatment option established so far (4, 5, 32). Diazepam and further benzodiazepines, as well as barbiturates, are often used to treat generalized tremor, but showed limited improvement in the present case as well as in former cases (14). The use of acepromazine appears to have improved the dog's condition in the present case. A study investigating the effect of the canine MDR1-mutation on sedation after IV administration of 0.04 mg/kg acepromazine, suggests that dogs with the homozygous MDR1-mutation are likely to show increased and prolonged sedation compared to normal dogs (33). These results indicate that CNS levels of acepromazine are expected to be increased in MDR1-mutant dogs and may explain the better effect of acepromazine compared to diazepam in the present case. However, the effect giving acepromazine IM and at higher doses, as described in the present case, has not yet been determined (33). Therefore, supportive care with fluid or even lipid infusion is the only treatment option so far. Consequently, further research analyzing the interaction of EMO with vertebrate CNS receptors would be essential to reach more specific treatment options in cases of neurological toxicity after EMO treatment.

## Conclusion

Despite strict recommendations and information in the SPC, the use of Profender® still leads to serious adverse drug reactions. Based on the current knowledge, two factors restrict the therapeutic safety of Profender®: drug application to non-fasted dogs and homozygous nt230(del4) MDR1 mutation (MDR1^−/−^). The combination of both factors probably explains the occurrence of neurological toxicity in the present case. Therefore, MDR1 genotyping in dogs from predisposed breeds as well as a strict fasting regime is recommended prior to Profender® administration. Additionally, it is essential to inform dog owners about these recommendations, in order to ensure appropriate drug application. Although the dog in the present case showed serious adverse reactions, these were transient and resolved without any specific treatment. To the best of our knowledge, there have not been any reports of cases with lethal outcome after the administration of Profender®. Nevertheless, studies elucidating the molecular target of EMO in the vertebrate brain are necessary to reach more specific treatment recommendations that would be needed in even more severe cases of adverse drug reactions after Profender® treatment.

## Consent for publication

Written informed consent was obtained from the owner for the publication of this case report and any potentially-identifying information.

## Author Contributions

CL clinically examined the dog and described the case. DG, MH, and JG discussed the case and drafted the manuscript. All authors reviewed, edited the manuscript, have read, and approved the final manuscript.

### Conflict of Interest Statement

The authors declare that the research was conducted in the absence of any commercial or financial relationships that could be construed as a potential conflict of interest.

## Abbreviations

CNS: central nervous system
EMO: emodepside
PZQ: praziquantel
MDR1: multidrug resistance gene 1
SPC: Summary of Product Characteristics.

## References

[1] Harder A von Samson-Himmelstjerna G. Cyclooctadepsipeptides–a new class of anthelmintically active compounds. Parasitol Res. (2002) 88:481–8. 10.1007/s00436-002-0619-2 12107468

[2] Krücken J Harder A Jeschke P Holden-Dye L O'Connor V Welz C. Anthelmintic cyclcooctadepsipeptides: complex in structure and mode of action. Trends Parasitol. (2012) 28:385-94. 10.1016/j.pt.2012.06.005 22858281

[3] Abongwa M Martin RJ Robertson AP. A brief review on the mode of action of antinematodal drugs. Acta Vet. (2017) 67:137–52. 10.1515/acve-2017-0013 29416226 PMC5798647

[4] EMA Profender: EPAR–Product Information. (2008). Available online at: https://www.ema.europa.eu/en/documents/product-information/profender -epar-product-information_en.pdf (accessed March 18, 2019).

[5] EMA Profender: EPAR–Scientific Discussion. (2008). Available online at: https://www.ema.europa.eu/en/documents/scientific-discussion/profender -epar-scientific-discussion_en.pdf (accessed March 18, 2019).

[6] Saeger B Schmitt-Wrede HP Dehnhardt M Benten WP Krücken J Harder A. Latrophilin-like receptor from the parasitic nematode Haemonchus contortus as target for the anthelmintic depsipeptide PF1022A. FASEB J. (2001) 15:1332–4. 10.1096/fj.00-0664fje 11344131

[7] Willson J Amliwala K Davis A Cook A Cuttle MF Kriek N. Latrotoxin receptor signaling engages the UNC-13-dependent vesicle-priming pathway in *C. elegans*. Curr Biol. (2004) 14:1374–9. 10.1016/j.cub.2004.07.056 15296755

[8] Guest M Bull K Walker RJ Amliwala K O'Connor V Harder A. The calcium-activated potassium channel, SLO-1, is required for the action of the novel cyclo-octadepsipeptide anthelmintic, emodepside, in *Caenorhabditis elegans*. Int J Parasitol. (2007) 37:1577–88. 10.1016/j.ijpara.2007.05.006 17583712

[9] Willson J Amliwala K Harder A Holden-Dye L Walker RJ. The effect of the anthelmintic emodepside at the neuromuscular junction of the parasitic nematode *Ascaris suum*. Parasitology. (2003) 126:79–86. 10.1017/s0031182002002639 12613766

[10] Bull K Cook A Hopper NA Harder A Holden-Dye L Walker RJ. Effects of the novel anthelmintic emodepside on the locomotion, egg-laying behaviour and development of *Caenorhabditis elegans*. Int J Parasitol. (2007) 37:627–36. 10.1016/j.ijpara.2006.10.013 17157854

[11] Epe C Kaminsky R. New advancement in anthelmintic drugs in veterinary medicine. Trends Parasitol. (2013) 29:129–34. 10.1016/j.pt.2013.01.001 23376212

[12] Elmshäuser S Straehle LC Kranz J Krebber R Geyer J. Brain penetration of emodepside is increased in P-glycoprotein-deficient mice and leads to neurotoxicosis. J Vet Pharmacol Ther. (2015) 38:74–9. 10.1111/jvp.12149 25131706

[13] Geyer J Döring B Godoy JR Leidolf R Moritz A Petzinger E. Frequency of the nt230 (del4) MDR1 mutation in Collies and related dog breeds in Germany. J Vet Pharmacol Ther. (2005) 28:545–51. 10.1111/j.1365-2885.2005.00692.x 16343287

[14] Elmshäuser S Wilke A Geyer A Drug intolerance after administration of emodepside/praziquantel (Profender) in MDR1-mutant dogs. Kleintierpraxis. (2014) 59:597–605. 10.2377/0023-2076-59-597

[15] Gramer I Leidolf R Döring B Klintzsch S Krämer EM Yalcin E. Breed distribution of the nt230(del4) MDR1 mutation in dogs. Vet J. (2011) 189:67–71. 10.1016/j.tvjl.2010.06.012 20655253

[16] Conder GA Johnson SS Nowakowski DS Blake TE Dutton FE Nelson SJ. Anthelmintic profile of the cyclodepsipeptide PF1022A in *in vitro* and *in vivo* models. J Antibiot. (1995) 48:820–3. 7592027 10.7164/antibiotics.48.820

[17] Harder A. Cyclooctadepsipeptides–an anthelmintically active class of compounds exhibiting a novel mode of action. Int J. Antimicrob Agents. (2003) 22:318–31. 10.1016/s0924-8579(03)00219-x 13678839

[18] Harder A Holden-Dye L Walker R Wunderlich F. Mechanisms of action of emodepside. Parasitol Res. (2005) 97(Suppl 1):S1–10. 10.1007/s00436-005-1438-z 16228263

[19] Holden-Dye L Crisford A Welz C von Samson-Himmelstjerna G Walker RJ O'Connor V. Worms take to the slo lane: a perspective on the mode of action of emodepside. Invert Neurosci. (2012) 12:29–36. 10.1007/s10158-012-0133-x 22539031 PMC3360863

[20] Holden-Dye L O'Connor V Hopper NA Walker RJ Harder A Bull K. SLO, SLO, quick, quick, slow: calcium-activated potassium channels as regulators of *Caenorhabditis elegans* behaviour and targets for anthelmintics. Invert Neurosci. (2007) 7:199–208. 10.1007/s10158-007-0057-z 17962986

[21] Harder A. The Biochemistry of haemonchus contortus and other parasitic nematodes. Adv Parasitol. (2016) 93:69–94. 10.1016/bs.apar.2016.02.010 27238003

[22] Miltsch SM Krücken J Demeler J Janssen IJ Krüger N Harder A. Decreased emodepside sensitivity in unc-49 gamma-aminobutyric acid (GABA)-receptor-deficient *Caenorhabditis elegans*. Int J Parasitol. (2012) 42:761–70. 10.1016/j.ijpara.2012.05.009 22727682

[23] Sasaki T Takagi M Yaguchi T Miyadoh S Okada T Koyama M. A new anthelmintic cyclodepsipeptide, PF1022A. J Antibiot. (1992) 45:692–7. 1624372 10.7164/antibiotics.45.692

[24] von Samson-Himmelstjerna G Harder A Schnieder T Kalbe J Mencke N *In vivo* activities of the new anthelmintic depsipeptide PF 1022A. Parasitol Res. (2000) 86:194–9. 10.1007/s004360050031 10726989

[25] Dornetshuber R Kamyar MR Rawnduzi P Baburin I Kouri K Pilz E. Effects of the anthelmintic drug PF1022A on mammalian tissue and cells. Biochem Pharmacol. (2009) 77:1437–44. 10.1016/j.bcp.2009.01.005 19426683

[26] Crisford A Murray C O'Connor V Edwards RJ Kruger N Welz C. Selective toxicity of the anthelmintic emodepside revealed by heterologous expression of human KCNMA1 in *Caenorhabditis elegans*. Mol Pharmacol. (2011) 79:1031–43. 10.1124/mol.111.071043 21415309 PMC3102553

[27] Crisford A Ebbinghaus-Kintscher U Schoenhense E Harder A Raming K O'Kelly I. The cyclooctadepsipeptide anthelmintic emodepside differentially modulates nematode, insect and human calcium-activated potassium (SLO) channel alpha subunits. PLoS Negl Trop Dis. (2015) 9:e0004062. 10.1371/journal.pntd.0004062 26437177 PMC4593646

[28] Frohberg H Schulze Schencking M. Toxicological profile of praziquantel, a new drug against cestode and schistosome infections, as compared to some other schistosomicides. Arzneimittelforschung. (1981) 31:555–65. 7195246

[29] EMEA Praziquantel: Summary Report (1)–Committee for Veterinary Medicinal Products. (1996). Available online at: https://www.ema.europa.eu/en/documents/mrl-report/praziquantel-summary -report-1-committee-veterinary-medicinal-products_en.pdf (accessed March 18, 2019).

[30] European Commission Volume 9 B of The Rules Governing Medicinal Products in the European Union–Guidelines onPharmacovigilanceforMedicinalProducts for Veterinary Use–Version October 2011. (2011). Available online at: https://ec.europa.eu/health/sites/health/files/files/eudralex/vol-9/vol_9b_2011-10.pdf (accessed July 03, 2019).

[31] European Commission Directive 2001/82/EC of the European Parliament and of the Council of 6 November 2001 on the Community Code Relating to Veterinary Medicinal Products. (2001). Available online at: https://eur-lex.europa.eu/legal-content/EN/TXT/PDF/?uri=CELEX:32001L0082&from=DE (accessed July 03, 2019).

[32] Karpstein T Pasche V Häberli C Scandale I Neodo A Keiser J. Evaluation of emodepside in laboratory models of human intestinal nematode and schistosome infections. Parasit Vectors. (2019) 12:226. 10.1186/s13071-019-3476-x 31088525 PMC6515646

[33] Deshpande D Hill KE Mealey KL Chambers JP Gieseg MA. The effect of the canine ABCB1-1Delta mutation on sedation after intravenous administration of acepromazine. J Vet Intern Med. (2016) 30:636–41. 10.1111/jvim.13827 26822006 PMC4913601

